# Therapeutic targeting of ARID1A-deficient cancer cells with RITA (Reactivating p53 and inducing tumor apoptosis)

**DOI:** 10.1038/s41419-024-06751-1

**Published:** 2024-05-29

**Authors:** Zihuan Wang, Xu Zhang, Yuchen Luo, Yijiang Song, Cheng Xiang, Yilin He, Kejin Wang, Yingnan Yu, Zhen Wang, Wenxuan Peng, Yi Ding, Side Liu, Changjie Wu

**Affiliations:** 1 Guangdong Provincial Key Laboratory of Gastroenterology, Department of Gastroenterology, Nanfang Hospital, Southern Medical University, Guangzhou, 510515 China; 2 Department of Radiation Oncology, Nanfang Hospital, Southern Medical University, Guangzhou, 510515 China; 3 Department of Laboratory Medicine, Nanfang Hospital, Southern Medical University, Guangzhou, 510515 China

**Keywords:** Targeted therapies

## Abstract

ARID1A, a component of the SWI/SNF chromatin-remodeling complex, is frequently mutated in various cancer types and has emerged as a potential therapeutic target. In this study, we observed that ARID1A-deficient colorectal cancer (CRC) cells showed synthetic lethal effects with a p53 activator, RITA (reactivating p53 and inducing tumor apoptosis). RITA, an inhibitor of the p53-MDM2 interaction, exhibits increased sensitivity in ARID1A-deficient cells compared to ARID1A wild-type cells. Mechanistically, the observed synthetic lethality is dependent on both p53 activation and DNA damage accumulation, which are regulated by the interplay between ARID1A and RITA. ARID1A loss exhibits an opposing effect on p53 targets, leading to decreased p21 expression and increased levels of proapoptotic genes, PUMA and NOXA, which is further potentiated by RITA treatment, ultimately inducing cell apoptosis. Meanwhile, ARID1A loss aggravates RITA-induced DNA damage accumulation by downregulating Chk2 phosphorylation. Taken together, ARID1A loss significantly heightens sensitivity to RITA in CRC, revealing a novel synthetic lethal interaction between ARID1A and RITA. These findings present a promising therapeutic approach for colorectal cancer characterized by ARID1A loss-of-function mutations.

## Introduction

Colorectal cancer (CRC) is the third most deadly and fourth most commonly diagnosed cancer worldwide [1]. Its incidence has steadily increased, especially in developing countries. According to the classic CRC carcinogenesis model, the initiation and progression of CRC require a spectrum of genetic mutations. The earliest trigger is APC inactivation [2]. Mutations in other suppressor genes (*SMAD2*, *SMAD4* [3], *DCC* [4], and *TP53* [5]), oncogenes (*KRAS* [6] and *BRAF* [7]), and other genes contribute to neoplastic transformation in foci of the altered colon mucosa, which in turn leads to further degeneration toward malignancy. Next-generation sequencing revealed that *ARID1A* (AT-rich interactive domain 1A) is one of the most frequently mutated genes in CRC [8]. As a subunit of the SWI/SNF chromatin-remodeling complex, which has been identified as the most commonly mutated chromatin modulator in human cancers, ARID1A is mutated in approximately 10% of patients with CRC [9]. Moreover, clinicopathological analyses revealed that ARID1A protein loss or reduced expression occurs in 77% of CRC samples [10]. As the tumor-node-metastasis (TNM) stage advanced, the proportion of ARID1A loss increased [10]. These data suggest that ARID1A is a key tumor suppressor in CRC and its loss is strongly linked to CRC progression and metastasis.

p53 is a critical tumor suppressor that plays a central role in the initiation and progression of cancer. As a transcription factor, it regulates the expression of genes involved in various cellular processes, such as the cell cycle, DNA repair, and cell apoptosis [11]. By driving these molecular processes, it inhibits cancer growth and safeguards genomic integrity, thus acting as a “guardian of the genome”. Its encoding gene *TP53* is mutated in more than half of all human cancers, including breast, colon, lung, liver, prostate, bladder, and skin [12]. Therefore, restoring the function of p53 in tumors is one of the most appealing anticancer therapeutic strategies. Several promising p53-based treatment approaches have emerged in recent years, and are currently undergoing clinical research [13]. MDM2, an E3 ubiquitin ligase, inhibits p53 by promoting its ubiquitination and proteasome-mediated degradation [14]. Therefore, MDM2 inhibitors can stabilize p53 and reactivate its function. Several small-molecule MDM2 inhibitors, such as idasanutlin (RG7388), APG-115, and AMG232, have entered clinical trials for cancer treatment [13]. Recently, through an open-label, first-in-human, phase Ia/Ib study in patients with advanced solid tumors, brigimadlin (BI 907828), an oral MDM2-p53 antagonist, showed encouraging preliminary efficacy (11.1% overall response and 74.1% disease control rates), particularly in patients with well-differentiated or dedifferentiated liposarcoma [15]. Although MDM2 inhibition is an appealing strategy, MDM2 inhibitors are not currently available clinically because of their serious side effects in normal tissues. Therefore, cancer-specific MDM2 inhibitors should be developed.

Given the high frequency of ARID1A loss or inactivation in cancer, the exploitation of anticancer therapeutics based on ARID1A status has attracted attention. Recent studies have shown that ARID1A has a synthetic lethal interaction with several cancer-associated proteins, including EZH2 [16], HDAC6 [17], GCLC [18], and AURKA [19]. Inhibiting these synthetic lethality targets results in selective vulnerabilities in ARID1A mutant OCCC [20], CRC [21], and breast cancer cells [22]. These studies demonstrate that synthetic lethal targeting of ARID1A is a promising approach for the development of novel cancer-targeted therapies. Based on this idea, we systematically screened an antitumor metabolic compound library to identify synthetic lethal compounds for ARID1A loss in CRC cells. We found that the small-molecule RITA (reactivates p53 and induces tumor apoptosis) exhibits selective vulnerability in CRC cells lacking ARID1A in vitro and in vivo. We further explored the mechanism by which ARID1A loss and RITA treatment converge on p53 activation and DNA damage accumulation, contributing to apoptosis in ARID1A-deficient CRC cells. This study identified RITA as a potential drug for the treatment of CRC with ARID1A loss.

## Materials and methods

### Cell lines and culture

HCT116 and RKO cells were obtained from the American Type Culture Collection (ATCC, Manassas, VA, USA). The HCT116 *ARID1A*^−^^/^^−^ cell line was generated using the CRISPR-Cas9 gene editing system, and the specific method was described in a previous publication. HCT116 cells were cultured in Gibco Roswell Park Memorial Institute (RPMI) 1640 medium supplemented with 10% fetal bovine serum (FBS) and 1% penicillin/streptomycin. RKO cells were cultured in Gibco Modified Dulbecco’s Eagle Medium (DMEM) supplemented with 10% FBS and 1% penicillin/streptomycin. All cell lines were cultured in a humidified incubator at 37 °C with 5% CO_2_. RITA (T1798) was purchased from TargetMol (Waltham, MA, USA).

### Anticancer metabolism compound library drug screening and cell viability measurement

The Anticancer metabolism compound library (L1200) containing 237 small-molecule inhibitors was purchased from TargetMol (MA, USA). Each compound was diluted by a gradient and arrayed in 384-well plates in an 8-dose inter-plate titration format, ranging from 0.02 to 50 μM. Then, HCT116 *ARID1A*^+/+^ or *ARID1A*^−^^/^^−^ #1 cells were both counted to 3000 cells and seeded per well in the 384-well plates containing working dilution of the compound library and incubated for 72 h at 37 °C CO_2_ incubator. When cell viability was measured, the cells were incubated with 10% Alamar Blue (Sigma-Aldrich, St. Louis, MO) in media solution for 3 h, and the fluorescence signal (ex560/em590) was read from the bottom of the plate using SpectraMax-M4 (Molecular Devices, Sunnyvale, CA). The IC50 values of each compound were calculated using GraphPad Prism 8.0 (GraphPad Software, La Jolla, CA, USA). Screening was performed in duplicate and the average IC50 value from the two screenings was used to identify synthetic lethality hits. SI was calculated using the following equation: SI = IC50 *ARID1A*^+/+^/IC50 *ARID1A*^−/−^. Compounds with an SI > 2 were selected as candidates for synthetic lethality.

### siRNA silencing of ARID1A

Two different ARID1A siRNAs (si*ARID1A* #1 and si*ARID1A* #2) were designed and synthesized by RiboBio (Guangzhou, China). The sequences were as follows: si*ARID1A* #1,5′-GGACCTCTATCGCCTCTAT-3′; si*ARID1A* #2,5′-GAAGCAGGCACCACTAACT-3′. siRNA transfection was performed using Lipofectamine 3000, and the procedure strictly followed the manufacturer’s instructions. Reverse transfection was performed to silence ARID1A in a 12-well plate. Briefly, after cells were trypsinized, 5 μL of siRNA was pipetted into 100 μL Opti medium to dilute to the indicated concentration and then mixed with 100 μL Opti medium containing 2 μL Lipofectamine 3000, and the transfection mixture was incubated at room temperature for 20 min. Cells were trypsinized and 1000 μL of cell suspension (1 × 10^5^ cells per well) was added to each well containing the transfection mixture. The cells were then incubated for 72 h in a CO_2_ incubator, and cell images were captured using U-HGLGPS (Olympus, Japan). ImageJ software was used to measure cell density and assess cell viability. The silencing efficiency was assessed by immunoblotting using an anti-ARID1A antibody.

### Western blot and antibodies

Whole-cell protein extracts were prepared using ice-cold RIPA buffer (25 mM Tris-HCl pH 7.6, 150 mM NaCl, 1% NP-40, 1% sodium deoxycholate, and 0.1% sodium dodecyl sulfate (SDS)). Each aliquot of protein sample was run on an SDS-polyacrylamide gel electrophoresis and transferred onto a nitrocellulose membrane for immunoblotting with primary antibodies, including ARID1A (Cell Signaling Technology, #12354S, 1:2000 dilution), Cleaved PARP (Cell Signaling Technology, #5625S, 1:2000 dilution), Cleaved caspase3 (Cell Signaling Technology, #9661S, 1:500 dilution), MDM2 (Cell Signaling Technology, #86934S, 1:1000 dilution), p53 (Cell Signaling Technology, #2527S, 1:2000 dilution), p21 (Cell Signaling Technology, #2947S, 1:2000 dilution), PUMA (Cell Signaling Technology, #98672S, 1:2000 dilution), NOXA (Cell Signaling Technology, #14766S, 1:2000 dilution), Phospho-ATM (Ser1981) (Cell Signaling Technology, #5883T, 1:1000 dilution), Phospho-ATR (Ser428) (Cell Signaling Technology, #2853T, 1:1000 dilution), Phospho-BRCA1 (Ser1524) (Cell Signaling Technology, #9009T, 1:1000 dilution), Phospho-Chk1 (Ser345) (Cell Signaling Technology, #2348T, 1:1000 dilution), Phospho-Chk2 (Thr68) (Cell Signaling Technology, #2197T, 1:1000 dilution), Phospho-p53 (Ser15) (Cell Signaling Technology, #9286T, 1:1000 dilution), Phospho-Histone H2A.X (Ser139) (Cell Signaling Technology, #9718T, 1:1000 dilution), MDM4 (Proteintech, #28747-1-AP, 1:1000 dilution), FANCD2 (Proteintech, # 28619-1-AP, 1:1000 dilution) and β-actin (Cell Signaling Technology, #3700S, 1:4000 dilution) antibodies, followed by horseradish peroxidase-conjugated secondary antibodies (ZEN-Bioscience, 511203). Uncropped versions of all blots are shown in Supplementary Figs. 6 and 7.

### Tumor xenograft mouse model

All animal procedures were approved by the Animal Research Ethics Committee of the Southern Medical University (SMUL2021076) and all animals received humane acre according to the criteria outlined in the ‘Guide for the Care and Use of Laboratory Animals’ (8th Edition). Eight-week-old female BALB/c nude mice were implanted with *ARID1A*^+/+^ (left flank) and *ARID1A*^−^^/^^−^ (right flank) HCT116 cells suspended in Matrigel. When both tumors were palpable, the mice were randomized into two groups (five mice per group) of equal tumor volumes for treatment with vehicle and RITA. However, researchers were not blinded to the groups during the experiments. Mice were treated with vehicle (sterile saline containing 5% dimethyl sulfoxide, 5% tween-80, and 5% polyethylene glycol-400) or RITA (10 mg kg^−1^, daily) via intravenous injection for 17 days. Tumor size was periodically measured with a Vernier caliper for 21 days, and tumor volume was calculated using the modified ellipsoid formula (long axis × short axis × short axis × 0.5). At the end of the experiment, the mice were sacrificed and the tumors were harvested for weighing and further analyses. The weights of mice were measured regularly during the drug injection period to assess potential drug toxicity.

### Cell cycle and apoptosis assays

Cell cycle and apoptosis assays were performed in accordance with the manufacturer’s instructions. The Cell Cycle Staining Kit (Multi Sciences, CCS012, CN) was used to assess the cell cycle, and the Annexin V FITC Apoptosis Detection Kit (Beyotime, C1062S, CN) was used to assess apoptosis. Briefly, for cell cycle analysis, cells were harvested, washed with PBS, and resuspended in 500 μl DNA staining solution containing 5 μl permeabilization solution for 30 min. For apoptosis analysis, cells were harvested, washed with PBS, and resuspended in 195 μl binding buffer containing 5 μl Annexin V FITC and 10 μl PI at room temperature for 20 min. Cell cycle and apoptosis data were acquired and analyzed using a Beckman Coulter flow cytometer (Beckman Coulter, California, US) and FlowJo v10 software (FlowJo LLC, USA), respectively.

### Coimmunoprecipitation

HCT116 cells were harvested using RIPA buffer (Fdbio Science, FD011, CN) containing protease inhibitors (NCM Biotech, P001, CN). Protein lysates were precleared by rotary incubation with 500 μl protein A/G magnetic beads (bimake, B23201, US) containing mouse normal IgG at 4 °C for 1 h. Then The supernatant was collected by magnetic separation, followed by rotary incubation with protein A/G beads and p53 antibody (Proteintech, 60283-2-Ig) at 4 °C overnight. The following day, protein A/G magnetic beads antibody-interacting protein compounds were separated from the protein lysates and washed gently three times with 1 ml RIPA buffer containing a protease inhibitor. Finally, the compounds were resuspended in 1× loading buffer and subjected to SDS-PAGE, followed by western blot analysis.

### Comet assay

DNA damage was assessed using a single-cell gel electrophoresis assay under neutral conditions with a DNA Damage Detection Kit (SCGE) (KeyGEN BioTECH, KGA240) according to the manufacturer’s protocol. Briefly, the normal melting point agarose (NMA) was spread on glass slides and solidified at 4 °C for 10 min. Cells were harvested 48 h after RITA treatment, mixed with low-melting-point agarose (LMA), and plated on glass slides with NMA as the second layer. The glass slides with agarose gel were placed in pre-cooled lysis buffer for 2 h at 4 °C, subjected to electrophoresis at 25 V for 30 min under alkaline conditions, and stained with PI. The presence of comet tails was determined using U-HGLGPS (Olympus, Japan). The tail moment was calculated as (percentage of DNA in the tail) × (tail length), where the percentage of DNA in the tail and tail length were quantified using the CASP software.

### RT-qPCR

Total RNA extraction and cDNA synthesis were performed using TRIzol (Thermo Fisher, #15596026) and the Evo M-MLV RT Premix kit (Accurate Biology, AG11706) according to the manufacturer’s instructions. Gene transcription levels were determined using the SYBR Green Pro Taq HS Premix kit (Accurate Biology, AG11701) with the primer sequences (Tsingke Biotechnology). CDKN1A primer sequences are as forward primer 5′-CGATGGAACTTCGAC TTTGTCA-3′ and reverse primer 5′-GCACAAGGGTACAAGACAGTG-3′. PUMA primer sequences are as: forward primer 5′-GCCAGATTTGTGAGACAAGAGG-3′ and reverse primer 5′-CAGGCACCTAATTGGGCTC-3′. NOXA primer sequences are as: forward primer 5′-ACCAAGCCGGATTTGCGATT-3′ and reverse primer 5′-ACTTGCACTTGTTCCTCGTGG-3′. FANCD2 primer sequences are as: forward primer 5′-GTTCGCCAGTTG GTGATGGAT-3′ and reverse primer 5′-GGGAAGCCTGTAACCGTGAT-3′.

### Statistical analysis

All data are presented as mean ± standard deviation (s.d.). Data analysis was performed using the GraphPad Prism 8 software (GraphPad Software, La Jolla, CA, USA). Differences between the control and test groups were analyzed using Student’s t-test and one-way analysis of variance (ANOVA), and statistical significance was set at P < 0.05.

## Results

### RITA induces a synthetic lethal effect in ARID1A-deficient cells

**Fig. 1:**
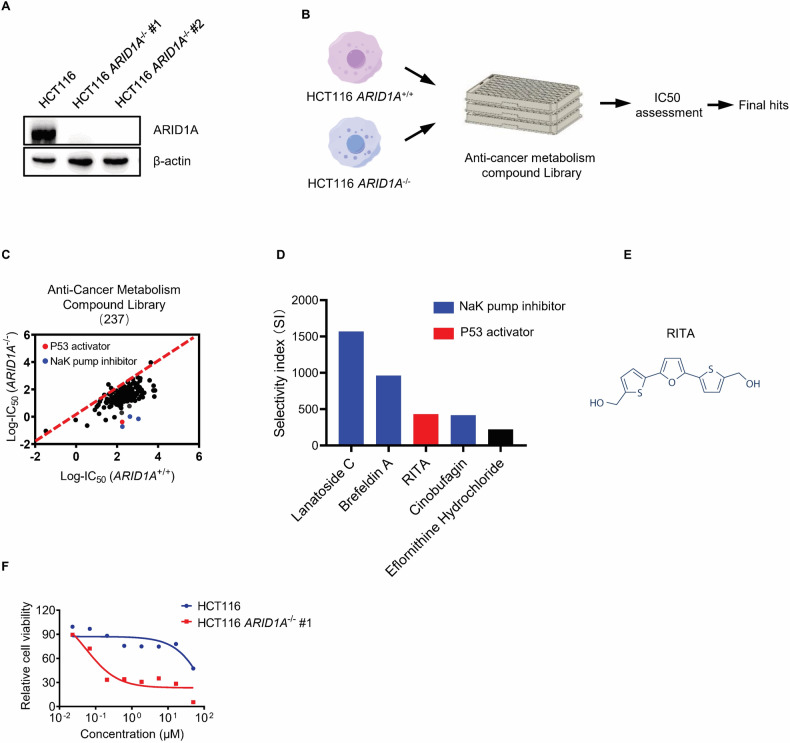
Screening of anticancer metabolism compound Library for synthetic lethality in HCT116 isogenic cells. **A** Loss of ARID1A expression in two HCT116 *ARID1A*^−^^/^^−^ clones was verified by western blot analysis. **B** Schematic illustration of the synthetic lethality screening procedure. HCT116 *ARID1A*^+/+^ and *ARID1A*^−^^/^^−^ #1 cell lines were screened in parallel with a 237 anticancer metabolism compound library in an 8-dose titration format. After incubation with the drug library for 72 h, cell viability was determined by Alamar Blue assay. **C** Scatter plot of the log2-IC50 for screening results. A log10 scale of IC50 values of the drugs against HCT116 *ARID1A*^+/+^ and *ARID1A*^−^^/^^−^ cells was plotted. **D** A bar graph depicting potential synergistic lethal compounds selected based on the selectivity index. SI = IC_50_^*ARID1A*(+/+)^/ IC_50_^*ARID1A*(^^−^^/^^−^^)^. Among all the compounds tested, the top 5 candidates are shown in the graph. **E** Structural formula of RITA. **F** Dose-response curves of HCT116 *ARID1A*^+/+^ and *ARID1A*^−^^/^^−^ #1 cell lines treated with RITA.

To identify ARID1A synthetic lethal targets in CRC, we used the ARID1A-isogenic HCT116 CRC pair, previously generated using the CRISPR/Cas9 system [19]. The ARID1A status was confirmed by immunoblot analysis (Fig. 1A). In agreement with previous reports, the growth rates of ARID1A-WT and ARID1A-deficient HCT116 CRC cells were largely similar in short-term culture [16, 19, 23, 24] (Supplementary Fig. 1). We then screened an anticancer metabolism compound library consisting of 237 cancer cellular metabolism-related compounds using ARID1A-isogenic cells. Screening was performed using an 8-dose titration, ranging from 0.02 to 50 μM, and the IC50 values of each compound were calculated using GraphPad software (Fig. 1B). From two rounds of screening, we identified five candidate drugs according to a selectivity index from top to bottom, including three sodium-potassium pump inhibitors (Lanatoside C, Brefeldin A, and Cinobufagin), one p53 activator (RITA), and one ornithine decarboxylase inhibitor (Eflornithine Hydrochloride) (Fig. 1C–F). Considering the importance of p53 in cancer, we selected the p53 activator RITA as the main synthetic lethal compound for ARID1A in CRC for the follow-up studies.

**Fig. 2:**
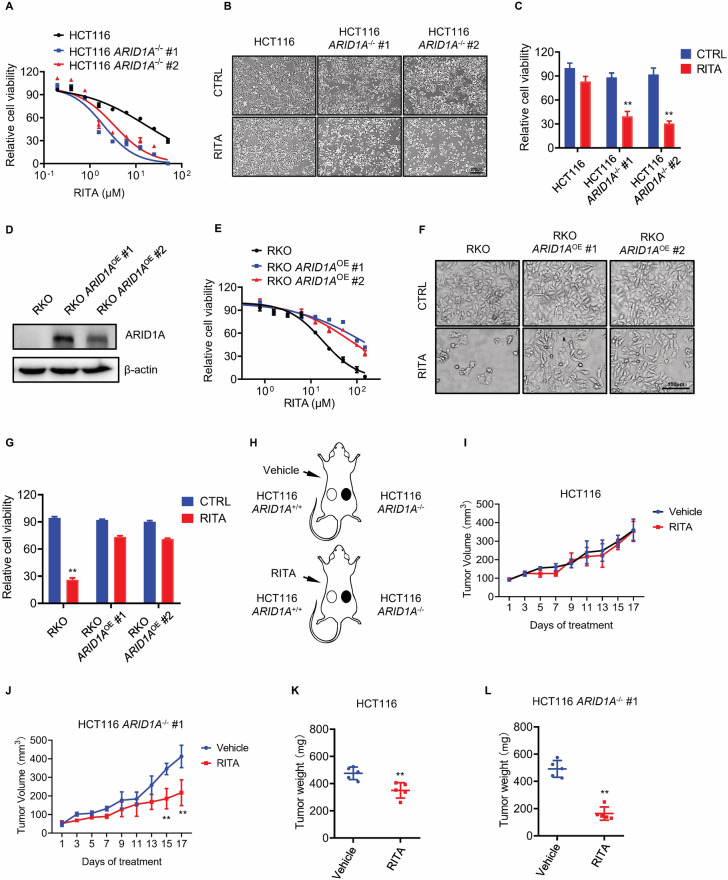
In vitro and in vivo synthetic lethality in HCT116 *ARID1A*^−^^/^^−^ cells by RITA. **A** Dose-response curves of HCT116 *ARID1A*^+/+^ and two *ARID1A*^−^^/^^−^ cell lines treated with RITA. HCT116 *ARID1A*^+/+^, *ARID1A*^−^^/^^−^ #1, and *ARID1A*^−^^/^^−^ #2 clones were incubated with RITA for 72 h and the cell viability was determined by Alamar Blue assay. Error bars represent s.d. (n = 3) from three independent experiments. **B**, **C** Synthetic lethality in HCT116 *ARID1A*^−^^/^^−^ cells by RITA. Representative images (**B**) and cell viability (**C**) are shown. HCT116 *ARID1A*^+/+^ and two *ARID1A*^−^^/^^−^ clones were treated with 150 nM RITA for 48 h and photographed under an Olympus microscope. Scale bar, 200 μm. Error bars represent s.d. from three independent experiments. ANOVA **P < 0.01. **D** Overexpression of ARID1A in two RKO *ARID1A*^OE^ clones was verified by western blot analysis. **E** Dose-response curves of RKO *ARID1A*^−^^/^^−^ and two RKO *ARID1A*^OE^ cell lines treated with RITA. RKO *ARID1A*^−^^/^^−^, *ARID1A*^OE^ #1, and *ARID1A*^OE^ #2 clones were incubated with RITA for 72 h and the cell viability was determined by Alamar Blue assay. Error bars represent s.d. (n = 3) from three independent experiments. **F**, **G** Synthetic lethality in RKO *ARID1A*^OE^ cells by RITA. Representative images (**F**) and cell viability (**G**) are shown. RKO *ARID1A*^−^^/^^−^ and two *ARID1A*^OE^ clones were treated with 20 μM RITA for 48 h and photographed under Olympus microscope. Scale bar, 100 μm. Error bars represent s.d. from three independent experiments. ANOVA **P < 0.01. **H** Schematic illustration of mouse tumor xenograft experiments with HCT116 *ARID1A*^−^^/^^−^ cell pair. **I**, **J** Tumor growth curve in nude mice bearing HCT116 *ARID1A*^+/+^ (**I**) or HCT116 *ARID1A*^−^^/^^−^ (**J**) xenografts after injection of vehicle or 10 mg kg^−1^ (mpk) RITA. Error bars represent **P < 0.01 between vehicle and RITA treatment groups (n = 5). Student’s *t*-test. **K**, **L** Wet weight measurement of the tumors isolated from mice bearing HCT116 *ARID1A*^+/+^ (**K**) or HCT116 *ARID1A*^−^^/^^−^ (**L**) xenografts at 21 days after injection of vehicle, 10 mpk RITA. Error bars represent s.d.

To further investigate the synthetic lethal effects of RITA and ARID1A, killing curves and cell morphology experiments were conducted in two ARID1A knockout (*ARID1A*^−^^/^^−^) clones. The results showed that RITA had a higher inhibition efficiency in the two *ARID1A*-KO clones than in *ARID1A*-WT (*ARID1A*^+/+^) cells (Fig. 2A–C). The IC50 value in *ARID1A*-WT cells was 16.7 μM. In two *ARID1A*-KO clones, the IC50 values are 2.0 μM and 3.4 μM, representing an 8.4-fold and 4.9-fold change, respectively. To further validate this synthetic lethal effect, we established another isogenic cell pair derived from the RKO CRC cell line, which has a frameshift mutation in ARID1A. Therefore, we reintroduced wild-type *ARID1A* into RKO CRC cells via lentivirus transfection and generated another ARID1A-isogenic cell pair (Fig. 2D). Consistent with the HCT116 isogenic cell pair, a synthetic lethal effect was observed (Fig. 2E–G). The IC50 value in the RKO cells was 18.2 μM. In two RKO *ARID1A*^OE^ clones, the IC50 values are 129.7 μM and 73.4 μM, representing a 4.5-fold and 2.5-fold change (Fig. 2E). These findings suggest that RITA treatment results in synthetic lethality, with ARID1A loss in CRC cells in vitro. In addition, we investigated the synthetic lethality of RITA in vivo using a tumor xenograft mouse model. Mice with ARID1A-isogenic tumors on either flank were intraperitoneally injected with RITA and tumor volumes were periodically measured (Fig. 2H). According to the tumor weight, RITA inhibited tumor growth with or without ARID1A expression. However, at the same dosage, ARID1A loss enhanced RITA-induced cell growth inhibition, which was in line with our in vitro findings (Fig. 2I–L and Supplementary Fig. 2). Daily administration of 10 mpk RITA did not appear to cause toxicity in mice, as assessed by changes in body weight (Supplementary Fig. 2A). In summary, these results collectively demonstrate that RITA treatment induces synthetic lethality in ARID1A-deficient CRC cells, both in vitro and in vivo.

### ARID1A deficiency sensitizes to RITA-induced cell apoptosis

**Fig. 3:**
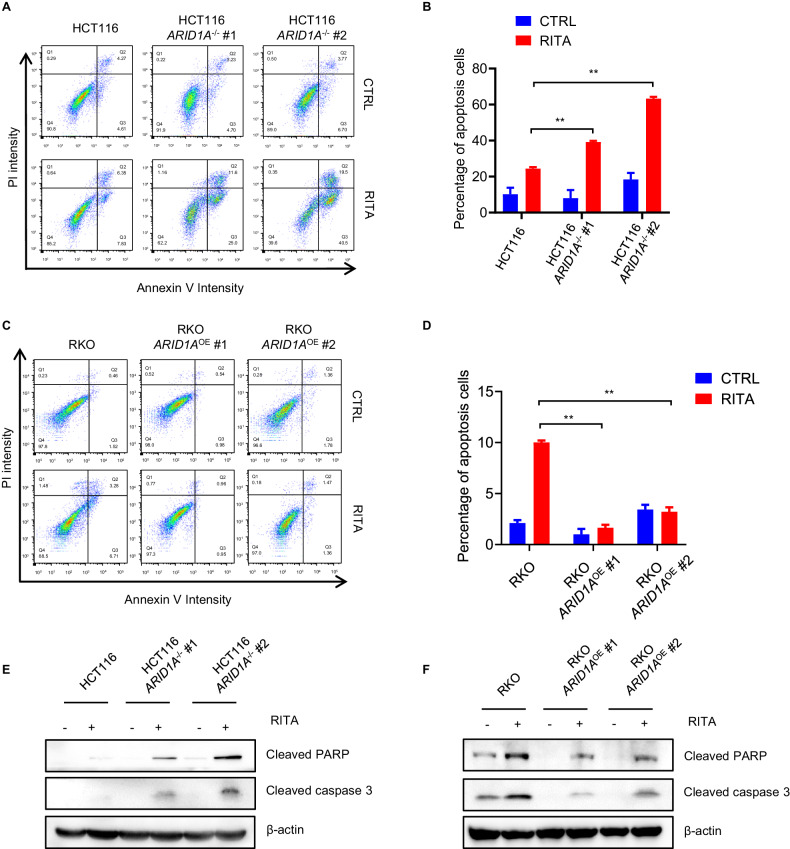
ARID1A deficiency sensitizes cells to RITA-induced apoptosis. **A**, **B, E** Apoptosis induced by RITA in HCT116 *ARID1A*^+/+^ and two *ARID1A*^−^^/^^−^ cell lines. Cells were treated with or without 100 nM RITA for 48 h and detected by flow cytometry with Annexin V/ PI staining (**A**) and quantitative analyses of apoptosis were performed (**B**). In addition, apoptotic biomarkers (e.g. Cleaved PARP, Cleaved caspase3) were measured with western blot analysis (**E**). **C**, **D, F** Apoptosis induced by RITA in RKO *ARID1A*^−^^/^^−^ and two RKO *ARID1A*^OE^ cell lines. Cells were treated with or without 10 μM RITA for 48 h and detected by flow cytometry with Annexin V/PI staining (**C**) and quantitative analyses of apoptosis were performed (**D**). Furthermore, apoptosis-related proteins were examined (**F**). Error bars represent s.d. from three independent experiments. ANOVA **P < 0.01.

RITA was initially described as an activator of p53. By binding to serine 33 and serine 37 sites of p53, RITA prevents its interactions with MDM2 and MDM4 [25], leading to elevated levels of p53 protein, cell growth arrest, and cell apoptosis in tumor cells of a different origin in vitro and in vivo [26]. Therefore, we analyzed the phenotype of CRC cells following RITA treatment. Following RITA treatment, flow cytometric analysis revealed a significant increase in apoptosis in *ARID1A*^−/−^ HCT116 cells (Fig. 3A, B). Similar to HCT116 cells, RITA treatment selectively increased apoptosis in ARID1A-deficient RKO cells (Fig. 3C, D). These results are consistent with those of the immunoblot analysis. Cell apoptosis markers (Cleaved PARP and Cleaved caspase3) were specifically increased in ARID1A-deficient cells both in HCT116 and RKO cells (Fig. 3E, F). Taken together, these results suggest that ARID1A deficiency sensitizes RITA-induced cell apoptosis, whereas its overexpression provides a protective effect.

### ARID1A loss has an opposing effect on p53 targets in CRC cells

**Fig. 4:**
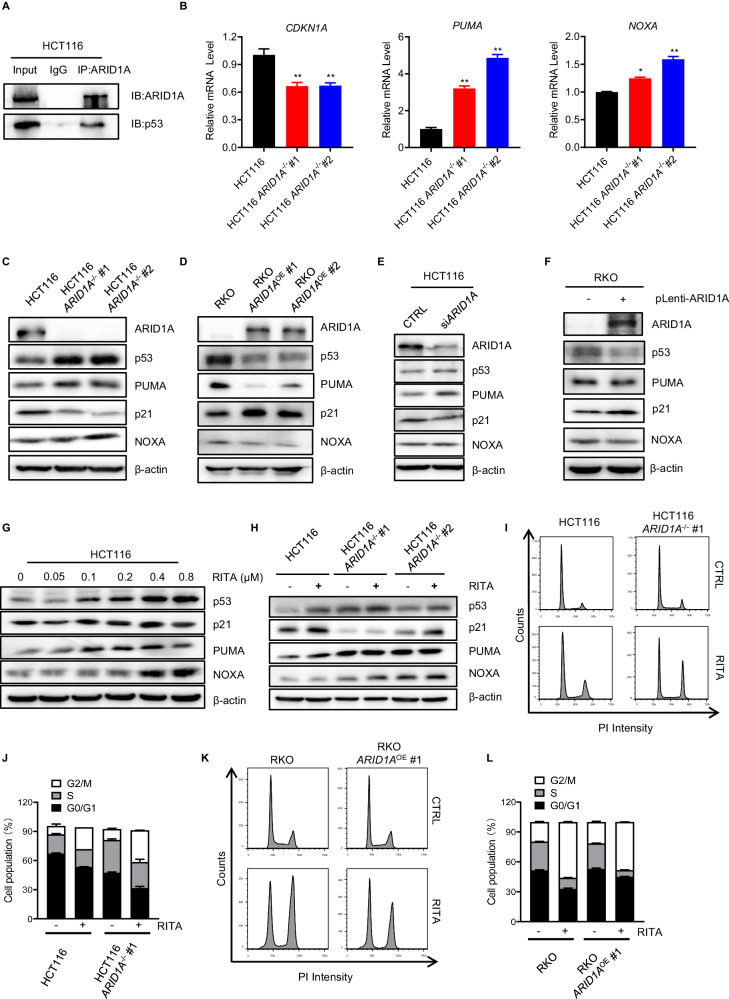
ARID1A and RITA cooperatively regulate the transcription of p53 target genes. **A** Interaction between ARID1A and p53. Coimmunoprecipitation assays were performed in HCT116 cell lines as described in “Methods” section. Input (cell lysates without immunoprecipitation) and IgG served as positive and negative controls, respectively. **B** RT-qPCR analysis of *CDKN1A*, *PUMA*, and *NOXA* mRNA level in HCT116 *ARID1A*^+/+^ and *ARID1A*^−^^/^^−^ clones. **P < 0.01, one-sample *t*-test. **C** Upregulation of p53, PUMA, and NOXA levels and downregulation of p21 level in HCT116 *ARID1A*^−^^/^^−^ cell lines. **D** Downregulation of p53, PUMA, and NOXA levels and upregulation of p21 level in RKO *ARID1A*^OE^ cell lines. **E** Upregulation of p53, PUMA, and NOXA levels and downregulation of p21 level by *ARID1A* silencing. **F** Downregulation of p53, PUMA, and NOXA levels and upregulation of p21 level by *ARID1A* overexpression. **G** Upregulation of p53, p21, PUMA, and NOXA levels with the increasing concentration of RITA treatment for 48 h in HCT116 cells. **H** Upregulation of p53, p21, PUMA, and NOXA level by 100 nM RITA treatment for 48 h in HCT116 *ARID1A*^+/+^ and *ARID1A*^−^^/^^−^ cell lines. **I**, **J** Effect of RITA on the cell cycle progression of HCT116 *ARID1A*^−^^/^^−^ cells. Cells were treated with 100 nM RITA for 48 h before flow cytometry. Cell cycle distribution (**I**) and cell population quantification (**J**) are shown. Error bars represent s.d. (*n* = 3 independent experiments). ANOVA **P < 0.01. **K**, **L** Effect of RITA on the cell cycle progression of RKO *ARID1A*^−^^/^^−^ cells. Cells were treated with 10 μM RITA for 48 h before flow cytometry. Cell cycle distribution (**K**) and cell population quantification (**L**) are shown. Error bars represent s.d. (*n* = 3 independent experiments). ANOVA **P < 0.01.

To study the mechanisms underlying synthetic lethality, we investigated the interplay between ARID1A and p53 in CRC cells. *ARID1A* and *TP53* are among the most frequently mutated tumor suppressor genes in cancers. Previous studies have shown that mutant *ARID1A* and *TP53* are mutually exclusive in a variety of cancers, including ovarian clear cells, uterine endometrioid carcinomas, and gastric, breast, and esophageal cancers [27]. These data indicated that ARID1A and TP53 collaborate to regulate cell progression. However, the role of ARID1A in the regulation of p53 target genes remains unclear. To address this, we examined the interaction between ARID1A and p53 using immunoprecipitation in CRC cells. This result demonstrated that in colorectal cancers, there was an interaction between ARID1A and p53 (Fig. 4A). Interestingly, we observed that ARID1A knockout increased the p53 protein levels. Similar to p53, MDM2, and MDM4 protein levels were upregulated in ARID1A-deficient cells (Supplementary Fig. 3). Meanwhile, in ARID1A-deficient cells, there was a reduced level of p21 but elevated levels of PUMA and NOXA, all of which are well-known p53 targets (Fig. 4B, C). Therefore, we hypothesized that the interaction between ARID1A and p53 might have an opposing effect on p53 target genes. This interaction can sustain p21 transcription levels but inhibits PUMA and NOXA levels. To test this hypothesis, we investigated the effect of ARID1A on p53 target genes in detail. Stable overexpression of wild-type ARID1A in ARID1A-deficient RKO cells resulted in an increase in p21 expression but decreased expression of PUMA and NOXA (Fig. 4D). To investigate whether target gene regulation by ARID1A was due to a secondary effect during long-term cellular adaptation following ARID1A deficiency, we tested the effect of transient depletion or overexpression of ARID1A on target gene expression. Transient depletion of ARID1A using siRNA effectively downregulated p21 expression and upregulated PUMA and NOXA expression (Fig. 4E). Furthermore, transient overexpression of wild-type ARID1A in RKO cells increased p21 expression and inhibited PUMA and NOXA expression (Fig. 4F). These results implied that ARID1A-deficient CRC cells had downregulated p21 and upregulated PUMA and NOXA, which potentially promoted apoptosis in this cell type. RITA, an activator of p53, can lead to elevated p53 levels and promote target gene expression. The effect was investigated using dose-response RITA treatment. There was a marked increase in p53 protein levels, along with upregulation of p21 and NOXA, as the RITA concentration increased (Fig. 4G). Next, we investigated the combined effects of ARID1A deficiency and RITA treatment on HCT116 ARID1A-isogenic CRC cell pairs. RITA treatment increased PUMA and NOXA levels in ARID1A-WT HCT116 cells and this effect was significantly enhanced in ARID1A-KO HCT116 cells (Fig. 4H). We further showed that RITA treatment induced S and G2/M phase arrest, especially in ARID1A-deficient cells both in HCT116 and RKO isogenic cell pairs (Fig. 4I–L). These results support the hypothesis that ARID1A protects cells from p53-induced cell apoptosis. Therefore, ARID1A loss increased RITA-induced apoptosis by promoting PUMA and NOXA expression.

### ARID1A loss enhances RITA-induced DNA damage

**Fig. 5:**
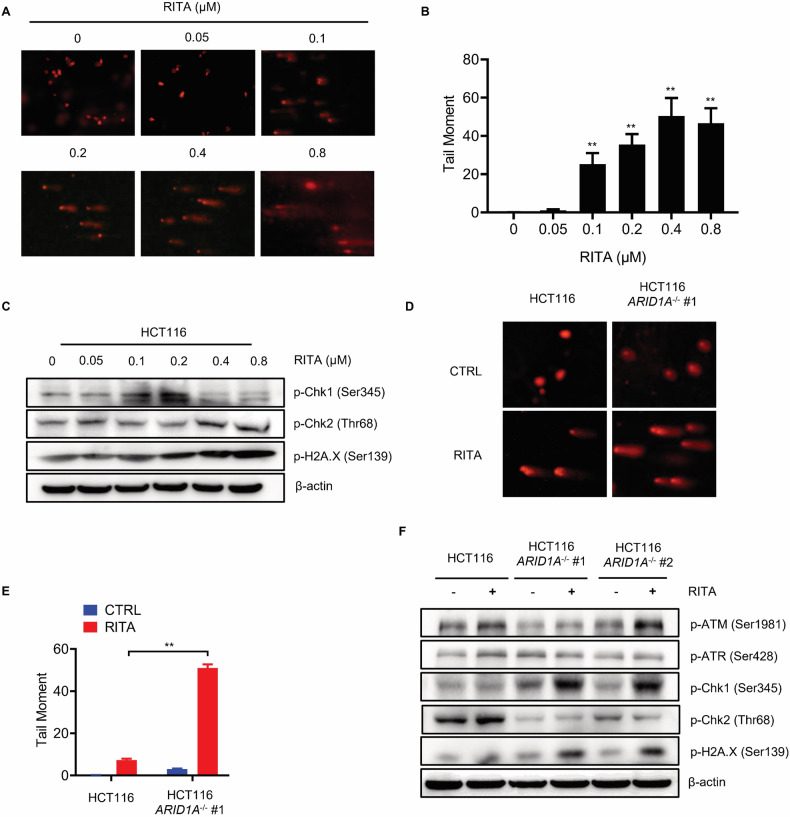
RITA treatment induces DNA Damage, particularly in ARID1A-deficient cells. **A** Representative cell nucleus fluorescent image and **B** quantification of the tail moment of HCT116 cells after RITA treatment. Cells were treated with an increasing concentration of RITA for 48 h. Error bars represent s.d. from three independent experiments. ANOVA **P < 0.01. **C** Upregulation of p-Chk1, p-Chk2, and p-H2A.X levels with the increasing concentration of RITA treatment for 48 h in HCT116 cells. **D** Representative cell nucleus fluorescent image and **E** quantification of the tail moment of HCT116 *ARID1A*^+/+^ and *ARID1A*^−^^/^^−^ cell lines after RITA treatment. Cells were treated with 150 nM RITA for 48 h. Error bars represent s.d. Student’s *t*-test. **F** Upregulation of p-Chk1, p-Chk2, and p-H2A.X levels in HCT116 ARID1A^+/+^ and ARID1A^−^^/^^−^ cell lines after 100 nM RITA treatment for 48 h.

Although the most widely accepted mechanism of RITA is the interaction between p53-MDM2 complex formation, recent work indicates that it can induce DNA damage in a p-chk2-dependent manner [28]. Considering that ARID1A is involved in repairing damaged DNA through homologous recombination (HR) repair [29], we hypothesized that ARID1A loss could enhance RITA-induced DNA damage. To test this hypothesis, we first investigated the effects of RITA on DNA damage accumulation. RITA treatment increased DNA damage as indicated by the comet assay (Fig. 5A, B). As long as the RITA concentration increased, DNA damage accumulated, which was also indicated by p-Chk1, p-Chk2, and p-H2AX levels (Fig. 5C). Interestingly, according to the results, RITA mainly induced single-strand DNA damage at low concentrations but double-stranded DNA damage at high concentrations. Next, we investigated the combined effects of ARID1A deficiency and RITA treatment on DNA damage in the ARID1A-isogenic CRC cell lines. RITA treatment-induced DNA damage, and this effect was significantly enhanced in *ARID1A*^−^^/^^−^ HCT116 cells (Fig. 5D, E). Similarly, p-Chk1 and p-H2AX levels were evaluated in *ARID1A*-KO cells (Fig. 5F). Interestingly, p-Chk2 levels were not upregulated in ARID1A-deficient cells following RITA treatment. Cellular responses to DNA damage are primarily coordinated by two distinct kinase signaling cascades, the ATM-Chk2 and ATR-Chk1 pathways, which are activated by DNA double-strand breaks (DSBs) and single-stranded DNA, respectively [30]. After RITA treatment, activation of p-ATM, p-ATR, and p-Chk2 was observed in HCT116 cells, especially in the ATM-Chk2 pathway, indicating that RITA mainly induced double DNA DSBs. The DSBs induced by RITA can be repaired by the ATM-Chk2 pathway at low concentrations, leading to limited Chk1 activation. However, according to the results, ARID1A is necessary for the activation of Chk2. ARID1A loss blocks Chk2 activation, which makes cells rely on Chk1 to repair RITA-induced DNA damage. Chk1 activation was observed in ARID1A-deficient cells following RITA treatment. The loss of function of Chk2 attenuated cell DNA damage repair ability, which is the reason why ARID1A loss induced more DNA damage and cell death after RITA treatment. Interestingly, it seems that Chk1/Chk2 activation is not induced by ATR/ATM, since there is no activation of ATR/ATM after RITA treatment, especially ATR. These results suggest that DNA damage repair is a common target of ARID1A and RITA and may play a key role in mediating synthetic lethality.

### The synthetic lethality between ARID1A and RITA is dependent on p53

**Fig. 6:**
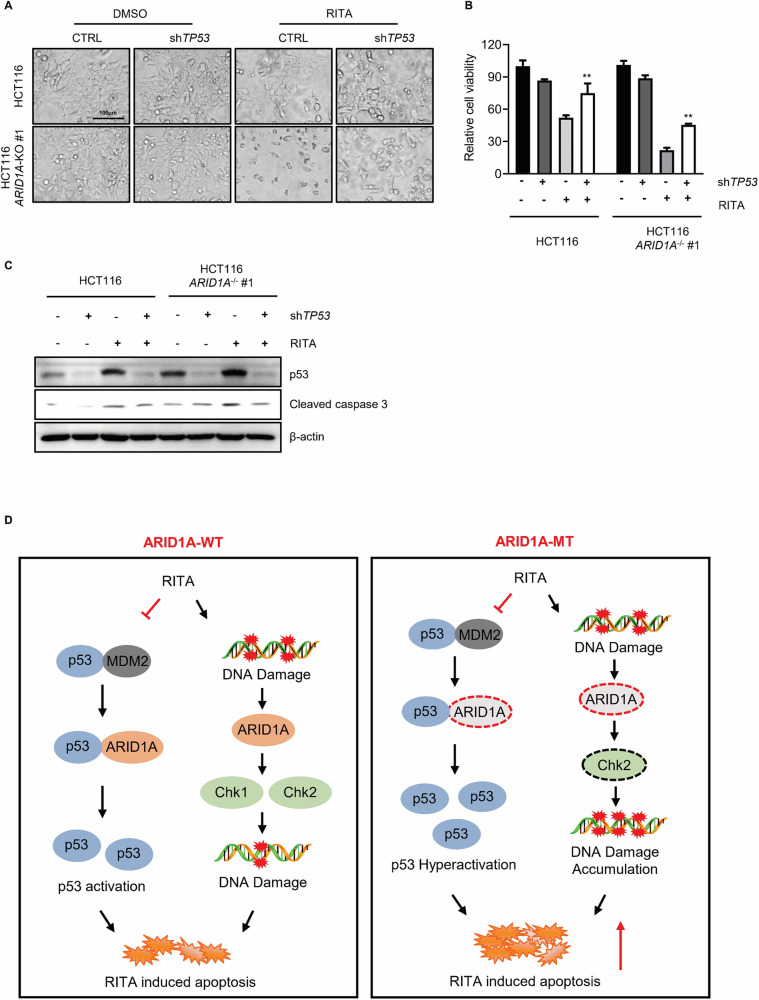
The synthetic lethality between ARID1A and RITA is dependent on p53. **A** Representative images and **B** cell viability of HCT116 *ARID1A*^+/+^ and *ARID1A*^−^^/^^−^ #1 cell lines with or without RITA and shTP53 treatment. Cells were treated with shTP53 for 24 h and later treated with 100 nM RITA for 48 h and photographed under Olympus microscope. Scale bar, 100 μm. Error bars represent s.d. from three independent experiments. ANOVA *P < 0.05. **C** Apoptotic biomarkers (e.g. Cleaved caspase3) were measured with western blot analysis. **D** Schematic diagram of the mechanism of synthetic lethality in HCT116 *ARID1A*^−^^/^^−^ cells by RITA.

We showed that RITA treatment promotes p53 and DNA damage accumulation, especially in ARID1A-deficient CRC cells, potentially contributing to the observed lethal effect. Recently, it was reported that a RITA analog, NSC782846, could induce cell death upon p53 reactivation but without inducing DNA damage, indicating the key role of p53 in RITA-induced cell death [31]. This result was validated in HCT116 *TP53* knockout cells. *TP53* knockout HCT116 cells were resistant to RITA treatment (Supplementary Fig. 4A, B). Therefore, to test whether the synthetic lethality between ARID1A and RITA is p53 dependent, we knocked down p53 with a shTP53 plasmid and examined synthetic lethality. p53 knockdown significantly reversed the inhibition of cell viability by RITA in HCT116 ARID1A-isogenic cells (Fig. 6A, B, and Supplementary Fig. 4C). Western blot analysis showed that p53 downregulation also reversed RITA-induced cell death (Fig. 6C). These results suggest that p53 contributes, at least in part, to the RITA-induced synthetic interactions with ARID1A in CRC cells. Considering that ARID1A participates in DNA damage repair by interacting with ATM/ATR, these results suggest that ARID1A-deficient cells have increased levels of p53 target proapoptotic genes and decreased DNA damage repair ability (Fig. 6D). These alterations are likely to facilitate selective vulnerability induced by RITA treatment in ARID1A-deficient cells.

FANCD2 is key in the Fanconi Anemia (FA) pathway, which plays a crucial role in DNA repair [32]. More importantly, RITA resistance is mediated by FANCD2 [33]. These results suggest that ARID1A loss potentially enhances RITA effectiveness through the regulation of FANCD2 expression. To test this hypothesis, we examined the transcription and protein levels of FANCD2 in ARID1A-deficient cells and found that ARID1A loss did not affect FANCD2 transcription, but FANCD2 protein levels showed a significant increase (Supplementary Fig. 3 and Supplementary Fig. 5A). This may be because ARID1A plays an important role in DNA damage repair, and when ARID1A is lost, cells rely on other mechanisms to repair DNA damage. In HCT116 *ARID1A*-WT and *ARID1A*-KO cell lines, no significant changes were observed after treatment (Supplementary Fig. 5B). These results indicated that FANCD2 may not be involved in the synthetic lethal effect induced by RITA in ARID1A-deficient cells. However, further experiments are needed to verify the FANCD2 function in this observed phenotype.

## Discussion

In this study, we observed that the small-molecule RITA selectively inhibited the growth of ARID1A-deficient CRC cells in vitro and of tumor xenografts in vivo. The observed synthetic lethality was attributed to both p53 overactivation and DNA damage accumulation in ARID1A-deficient cells. We found that ARID1A has an opposing effect on p53 targets. The loss of ARID1A decreased p21 transcription; however, conversely, by releasing p53 from the ARID1A-p53 complex, ARID1A loss enhanced p53 transcriptional activity on proapoptotic genes, including PUMA and NOXA. This effect was further enhanced by RITA treatment. Additionally, ARID1A loss contributed to RITA-induced DNA damage by inhibiting Chk2 phosphorylation. In summary, our study reveals a novel synthetic lethal interaction between ARID1A and RITA. Considering that p53-MDM2 inhibitors have completed a phase II clinical trial [15], our results suggest a promising therapeutic avenue for *ARID1A*-deficient colorectal cancers.

ARID1A and TP53 are frequently mutated across cancers but rarely in the same primary tumor. Multiple studies in solid tumors have demonstrated an inverse relationship between TP53 and ARID1A mutations, and even found ARID1A and TP53 mutual exclusivity in ovarian clear cell and uterine endometrioid carcinomas, indicating many functional connections between these two genes [27]. It has been reported that in breast cancer, low ARID1A expression is associated with a higher percentage of p53(+) percentage [34, 35]. In contrast, in endometrioid carcinoma, there was no significant relationship between loss of ARID1A and p53 overexpression [36]. These results indicate that the relationship between ARID1A and p53 may differ depending on cancer type. In CRC, we observed an increase in p53 protein levels after ARID1A knockout, which indicates that there is a negative regulation of p53 by ARID1A in colorectal cancer, most probably indirect. It has been demonstrated that ARID1A interacts with p53 via the c-terminal region and stimulates transcriptional activity. In an ovarian cancer cell line model, ARID1A directly interacts with p53 to regulate the transcription of p53 target genes such as p21, leading to the induction of p21 and subsequent cell cycle arrest [37]. Here, we demonstrated that ARID1A loss-of-function has an opposing effect on p53 target genes. In colorectal cancer, loss of ARID1A leads to a decrease in p21 expression, which is further supported in gynecologic cancers [38]. However, for PUMA and NOXA, well-known p53 target proapoptotic genes, loss of ARID1A results in an increase in their protein levels. These results suggested that the interaction between ARID1A and p53 can either promote or decrease the transcription of p53 target genes. In ARID1A-deficient cells, RITA treatment further enhanced p53 activation, leading to overexpression of PUMA and NOXA and promotion of cell apoptosis. These data highlight the tight regulation of p53 protein levels in ARID1A-deficient cancer cells, as both loss of function and overexpression of p53 induce cell death.

As initially identified as a p53-interacting molecule, RITA was reported to bind to the p53 N-terminal domain, probably leading to conformational changes, thus impeding the p53/MDM2 interaction and resulting in p53 accumulation, tumor cell growth inhibition, and p53-dependent cell apoptosis in vitro and in vivo [39]. Interestingly, several recent studies suggest that questioning its selective binding to p53, RITA might not be a direct inhibitor of the p53-MDM2 interaction, but rather disrupt the interaction indirectly by triggering DNA damage signaling-dependent, ATM/CHK2-mediated phosphorylation of p53 [28]. Importantly, ARID1A is also involved in repairing damaged DNA through homologous recombination (HR) repair [40]. Mechanistically, ARID1A is recruited to the DNA DSB sites by interacting with the upstream kinase ATR. ARID1A also helps recruit the ATPase subunit of the SWI/SNF complex to DNA damage sites. Loss of ARID1A also impairs the G2/M DNA damage checkpoint. Overall, ARID1A facilitates DSB end resection and maintains checkpoint signaling. Therefore, the loss of function of ARID1A attenuates DNA repair. RITA-induced DNA damage can easily accumulate because of the attenuation of the DNA damage repair ability in ARID1A-deficient cells, resulting in DNA damage-induced cell death.

In summary, our findings indicate that the small-molecule RITA exhibits a synthetic lethal effect with ARID1A loss. RITA treatment triggers p53 activation and accumulation of DNA damage, ultimately causing cell death, especially in ARID1A-deficient cancer cells. Given the high mutation rate of the SWI/SNF complex and the significance of ARID1A within this complex, we anticipate that our findings will have broad implications in the development of therapeutic interventions for these malignancies.

### Supplementary information

Supplementary Data

## Data Availability

All data supporting this study are available from the corresponding author upon reasonable request.
