# Supplementary material for: Therapeutic targeting of ARID1A-deficient cancer cells with RITA (Reactivating p53 and inducing tumor apoptosis)
Source: Cell Death Dis. 2024 May 29;15(5):375. doi: 10.1038/s41419-024-06751-1 (PMC11136964; doi:10.1038/s41419-024-06751-1)
Supplement: Supplementary file 1 — Supplementary Data [file 41419_2024_6751_MOESM1_ESM.docx]

| 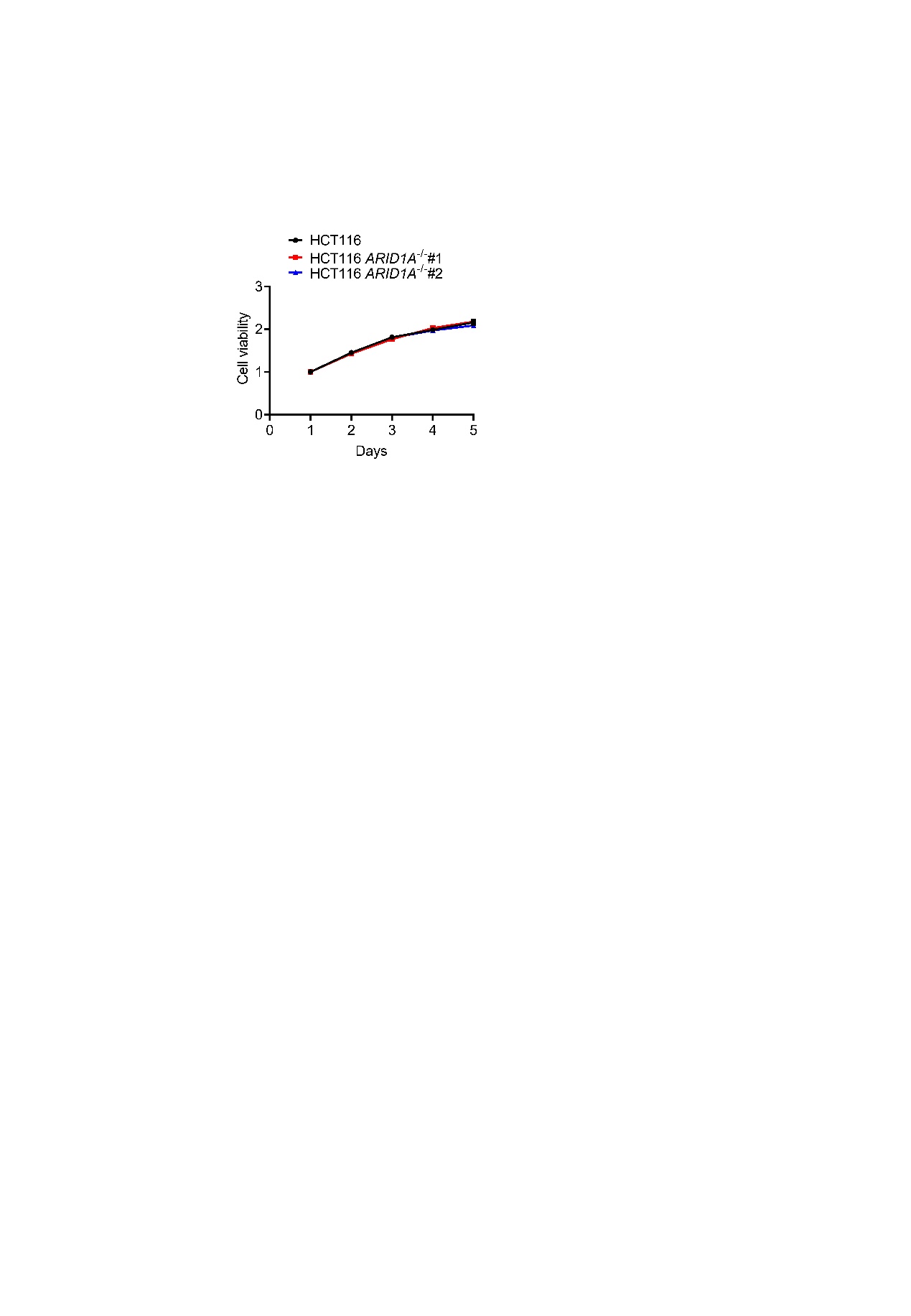 |
| --- |
| **Supplementary Fig. 1 Growth rate of ARID1A-isogenic colorectal cancer cells.** ARID1A-isogenic cell lines were grown in a 96-well plate until confluent and assessed for Alamar Blue assay. No significant difference was observed in growth rate between *ARID1A*-WT and KO cells. |

| 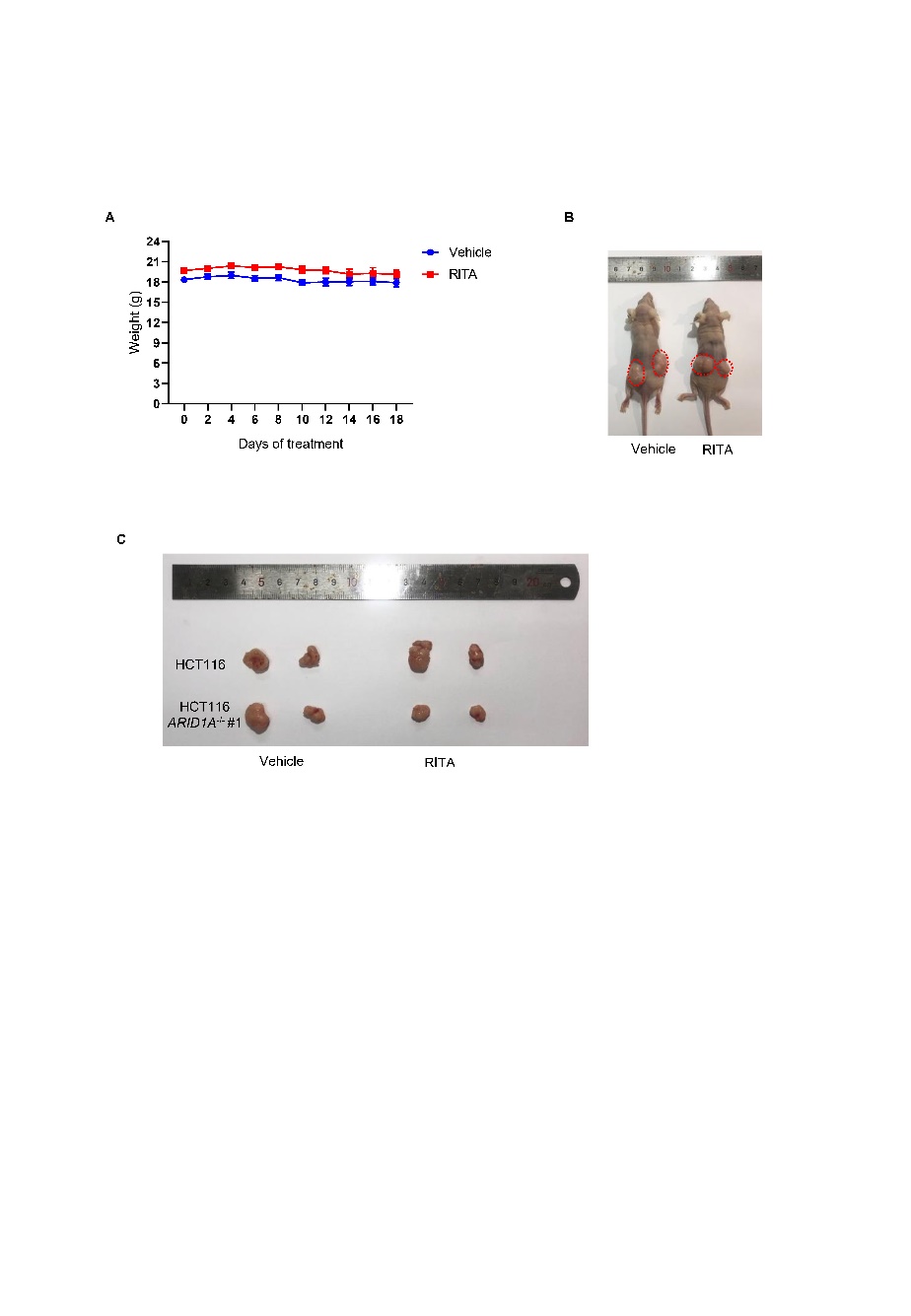 |
| --- |
| **Supplementary Fig. 2 ARID1A-isogenic CRC mouse xenograft model.** **A** Body weight of mice treated with RITA for 18 days. **B** Representative images of vehicle-treated and RITA-treated nude mice bearing HCT116 WT and *ARID1A*^-/-^ xenografts. **C** Representative images of tumor size with or without RITA treatment. |
| 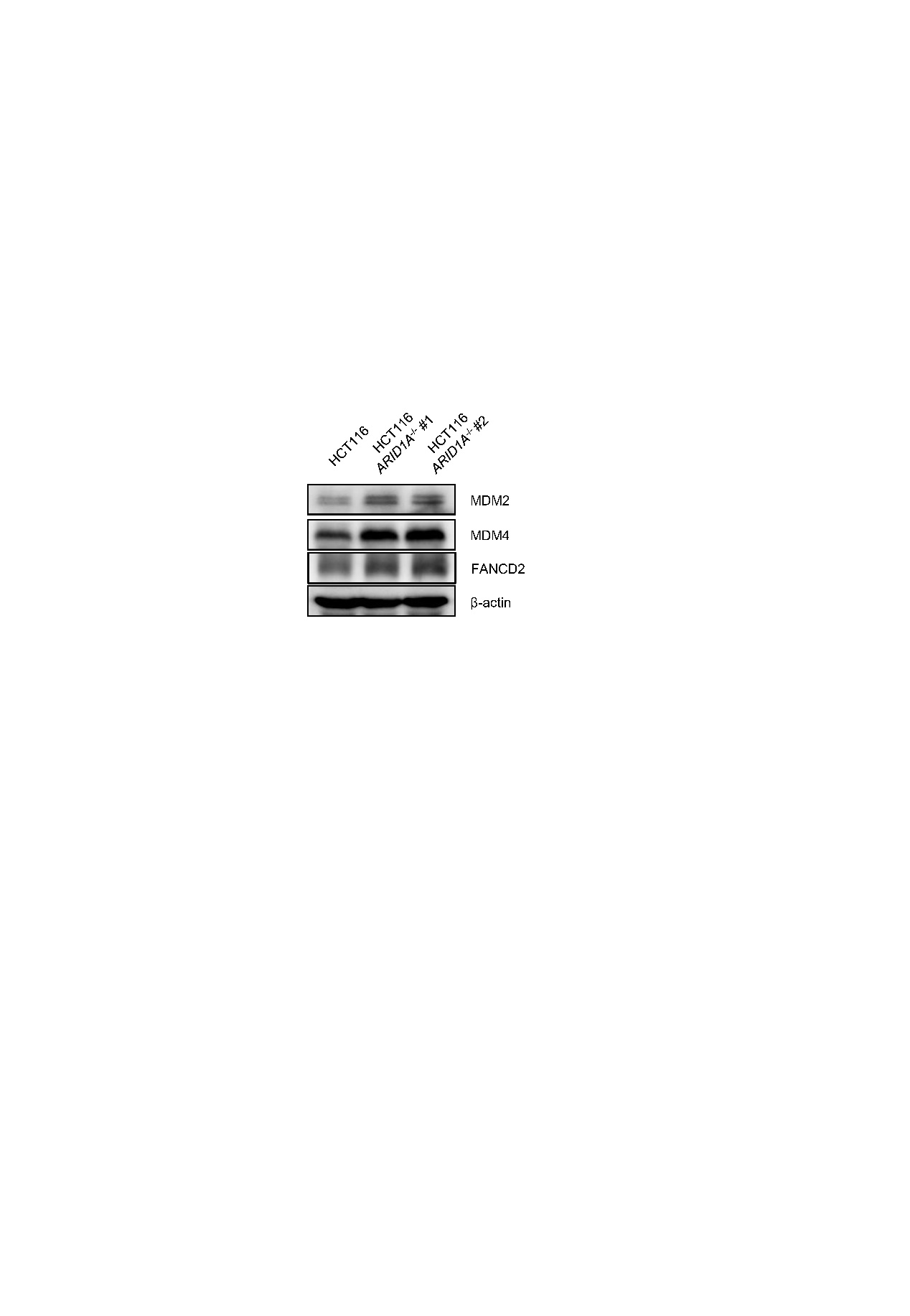 |
| **Supplementary Fig. 3 ARID1A negatively regulates MDM2, MDM4 and FANCD2.** Upregulation of MDM2, MDM4 and FANCD2 protein level in HCT116 *ARID1A*^-/-^ cell lines. |

| 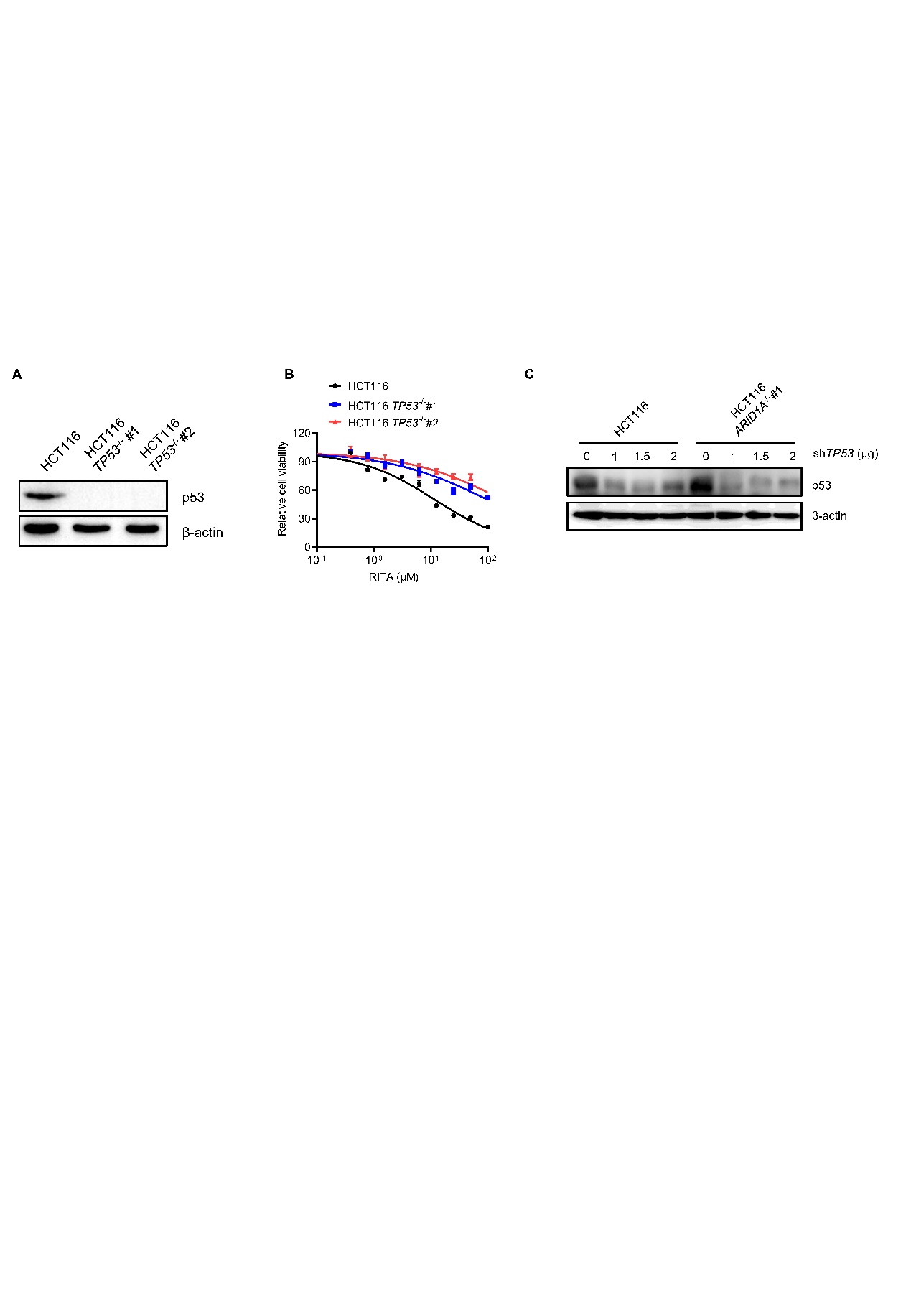 |
| --- |
| **Supplementary Fig. 4 *TP53* knockout makes cells resistant to RITA treatment.** **A** p53 status was verified with western blot in HCT116 *TP53*-WT and *TP53*-KO cells. **B** Dose-response curves of HCT116 *TP53*^+/+^ and two *TP53*^-/-^ cell lines treated with RITA for 72 h. **C** Knockdown efficiency of sh*TP53* was validated with western Blot in HCT116 and HCT116 *ARID1A*^-/-^ #1 cells. |

| 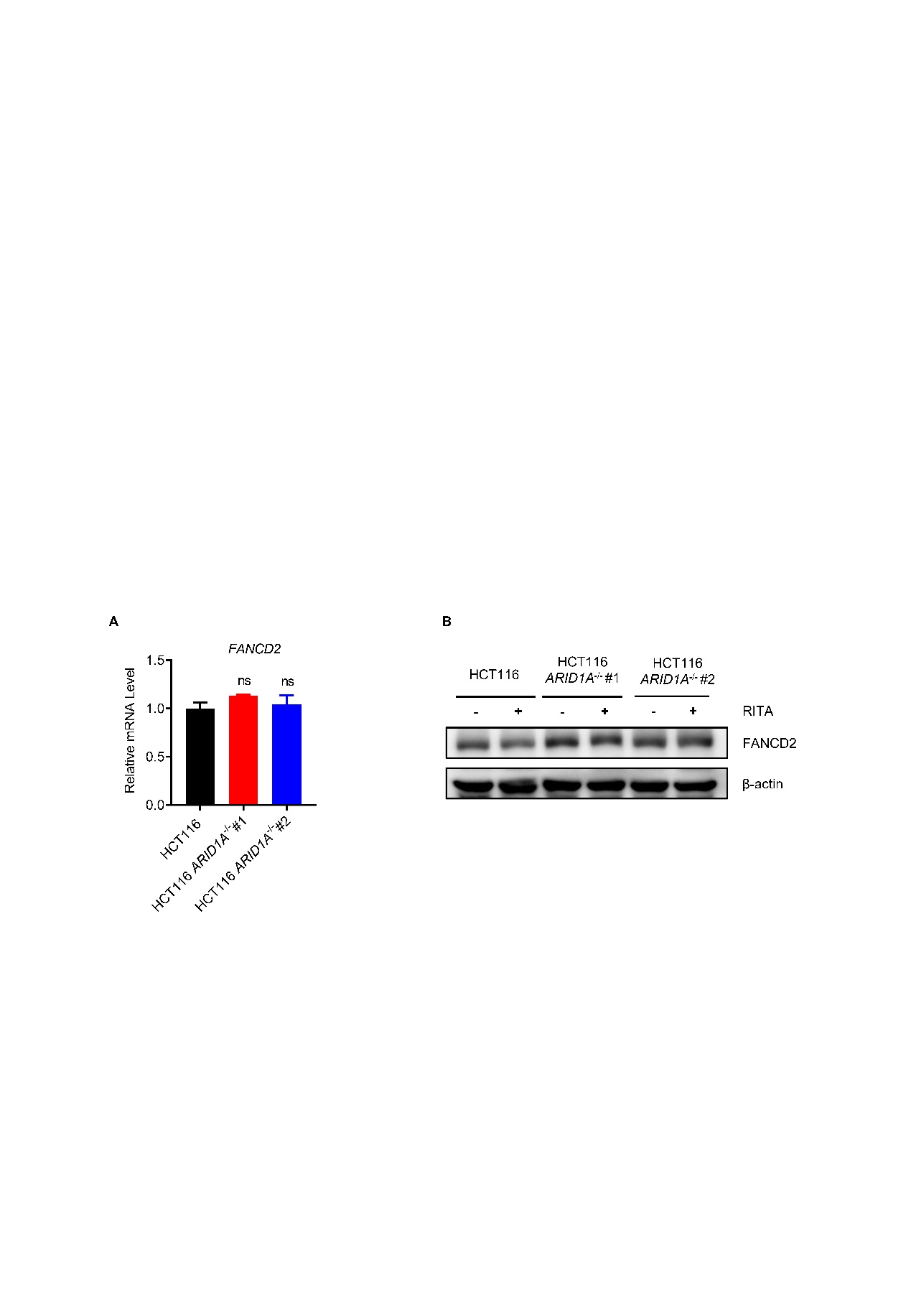 |
| --- |
| **Supplementary Fig. 5 Validation of FANCD2 expression. A** RT-qPCR analysis of FANCD2 mRNA level in HCT116 *ARID1A*^+/+^ and *ARID1A*^-/-^ clones. **B** FANCD2 status was verified with western blot in HCT116 and HCT116 *ARID1A*^-/-^ cells treated with or without 100 nM RITA for 48 h. |

| 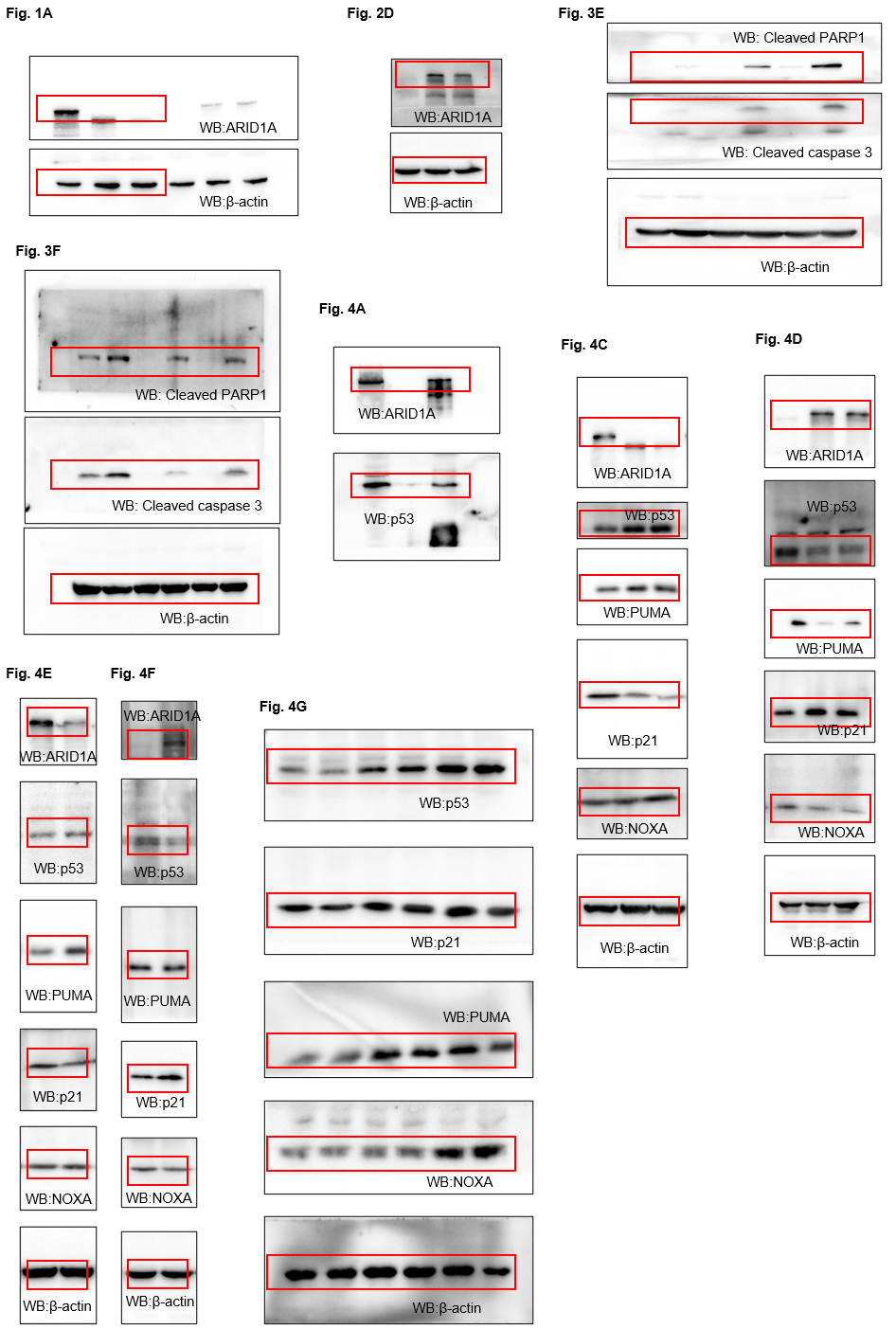 |
| --- |
| **Supplementary Fig. 6 Original Western Blot shown in Figures 1-4.** Each figure corresponds to the Western Blots in the indicated Figure number. |

| 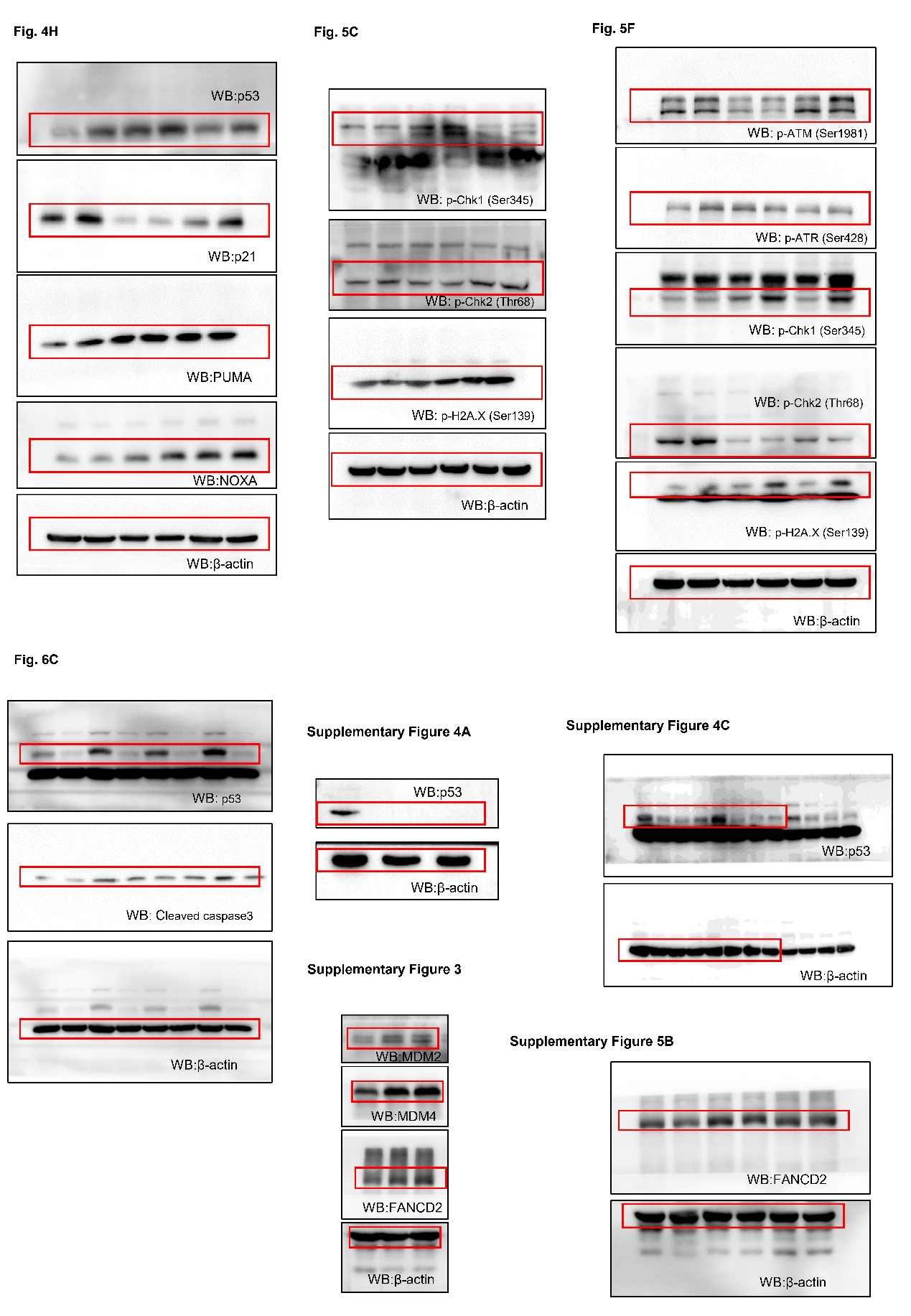 |
| --- |
| **Supplementary Fig. 7 Original Western Blot shown in Figures 4-6 and Supplementary Figures.** Each figure corresponds to the Western Blots in the indicated Figure number. |
